# Prion-like propagation of human brain-derived alpha-synuclein in transgenic mice expressing human wild-type alpha-synuclein

**DOI:** 10.1186/s40478-015-0254-7

**Published:** 2015-11-26

**Authors:** Maria E. Bernis, Julius T. Babila, Sara Breid, Katharina Annick Wüsten, Ullrich Wüllner, Gültekin Tamgüney

**Affiliations:** German Center for Neurodegenerative Diseases (DZNE), Sigmund-Freud-Straße 25, Gebäude 13, BMZ1, 53127 Bonn, Germany; Department of Neurology, University of Bonn, Sigmund-Freud-Straße 25, 53105 Bonn, Germany

**Keywords:** Parkinson’s disease, Multiple system atrophy, Incidental Lewy body disease, alpha-synuclein, Synucleinopathy, Prion-like

## Abstract

**Introduction:**

Parkinson’s disease (PD) and multiple system atrophy (MSA) are neurodegenerative diseases that are characterized by the intracellular accumulation of alpha-synuclein containing aggregates. Recent increasing evidence suggests that Parkinson’s disease and MSA pathology spread throughout the nervous system in a spatiotemporal fashion, possibly by prion-like propagation of alpha-synuclein positive aggregates between synaptically connected areas. Concurrently, intracerebral injection of pathological alpha-synuclein into transgenic mice overexpressing human wild-type alpha-synuclein, or human alpha-synuclein with the familial A53T mutation, or into wild-type mice causes spreading of alpha-synuclein pathology in the CNS. Considering that wild-type mice naturally also express a threonine at codon 53 of alpha-synuclein, it has remained unclear whether human wild-type alpha-synuclein alone, in the absence of endogenously expressed mouse alpha-synuclein, would support a similar propagation of alpha-synuclein pathology in vivo.

**Results:**

Here we show that brain extracts from two patients with MSA and two patients with probable incidental Lewy body disease (iLBD) but not phosphate-buffered saline induce prion-like spreading of pathological alpha-synuclein after intrastriatal injection into mice expressing human wild-type alpha-synuclein. Mice were sacrificed at 3, 6, and 9 months post injection and analyzed neuropathologically and biochemically. Mice injected with brain extracts from patients with MSA or probable iLBD both accumulated intraneuronal inclusion bodies, which stained positive for phosphorylated alpha-synuclein and appeared predominantly within the injected brain hemisphere after 6 months. After 9 months these intraneuronal inclusion bodies had spread to the contralateral hemisphere and more rostral and caudal areas. Biochemical analysis showed that brains of mice injected with brain extracts from patients with MSA and probable iLBD contained hyperphosphorylated alpha-synuclein that also seeded aggregation of recombinant human wild-type alpha-synuclein in a Thioflavin T binding assay.

**Conclusions:**

Our results indicate that human wild-type alpha-synuclein supports the prion-like spreading of alpha-synuclein pathology in the absence of endogenously expressed mouse alpha-synuclein in vivo.

**Electronic supplementary material:**

The online version of this article (doi:10.1186/s40478-015-0254-7) contains supplementary material, which is available to authorized users.

## Introduction

Parkinson’s disease (PD), multiple system atrophy (MSA), and dementia with Lewy bodies (DLB) are part of a spectrum of neurodegenerative disorders characterized by accumulation of misfolded alpha-synuclein, preferentially in cells of the nervous system, and are therefore often conceptualized as synucleinopathies [52, 53, 60]. Missense mutations in alpha-synuclein, such as A53T, or duplications and triplications of the *SNCA* gene encoding alpha-synuclein have been linked to familial PD and suggest that in synucleinopathies alpha-synuclein itself can become pathogenic [4, 15, 20, 37, 51, 62]. This has also raised interest in the putative relationship between posttranslational modifications of alpha-synuclein, such as hyperphosphorylation at S129, and its misfolding and deposition in disease [11, 49].

The seminal neuropathological analyses of Braak and co-workers suggest that the regional distribution of pathological alpha-synuclein in the brains of patients is progressive and can be classified into stages [3, 27]. Although this concept is not undisputed [35], aggregation of alpha-synuclein in embryonic dopamine neurons grafted into the striatum of PD patients for therapy spurred the hypothesis that misfolded alpha-synuclein may indeed spread between cells and seed aggregation of normal alpha-synuclein [17, 25]. Subsequent studies in cultured neurons and rodents showed that exogenous pathological alpha-synuclein can seed misfolding and aggregation of native alpha-synuclein and that pathological alpha-synuclein may propagate in a prion-like manner within the nervous system [8, 18, 24, 28, 29, 31, 33, 41, 42, 47, 58, 59, 61]. In most of the above studies either synthetic alpha-synuclein fibrils generated in vitro or transgenic M83-mice overexpressing alpha-synuclein with the familial A53T mutation, or wild-type mice or rats naturally expressing a threonine at codon 53 were used for inoculation experiments. While propagation of alpha-synuclein pathology can also be induced in M20-mice overexpressing human wild-type alpha-synuclein in the presence of endogenously expressed mouse alpha-synuclein [45, 46], it remains unresolved whether human wild-type alpha-synuclein by itself, in the absence of endogenously expressed mouse alpha-synuclein, would support prion-like spreading of pathological alpha-synuclein in mice. Moreover, it is unresolved whether endogenously expressed mouse alpha-synuclein may actually hamper misfolding and spreading of transgenically expressed human alpha-synuclein. In the field of prion diseases, transgenic mice expressing human prion protein in the presence of endogenously expressed mouse prion protein are resistant to infection with human prions from Creutzfeldt-Jakob disease patients, and only become susceptible to human prions when endogenous expression of the mouse prion protein is ablated [56, 57]. We therefore investigated the fate of mice expressing human wild-type alpha-synuclein on a knockout background for mouse alpha-synuclein after intrastriatal injection of brain extracts from patients with MSA and probable incidental Lewy body disease (iLBD) [14, 21]. Our results suggest that human wild-type alpha-synuclein indeed supports prion-like propagation of pathological alpha-synuclein from the brains of MSA patients. Moreover, our results suggest that not only MSA brains but also brains of individuals with iLBD contain misfolded alpha-synuclein species that can induce prion-like spreading of misfolded alpha-synuclein in mice.

## Materials and methods

### Preparation of brain extracts for injections

Insoluble fractions from human cerebral cortices were prepared as previously described [31]. Briefly, 0.2 g of frozen cortical brain tissue from two MSA and two probable iLBD cases (Table 1) each were homogenized in 18 volumes of Buffer A [10 mM Tris–HCl, pH 7.4, 0.8 M NaCl, 1 mM EGTA, 10 % sucrose, with protease and phosphatase inhibitors (cOmplete and PhosStop, Roche Diagnostics)] by three 60 s cycles in a Precellys 24-Dual homogenizer (Peqlab). Sarkosyl was added to the homogenate at a final concentration of 2 %. The mixture was incubated for 30 min at 37 °C, sonicated, and spun at 9,100 × *g* for 10 min. The supernatant was further centrifuged at 100,000 × *g* for 30 min at 25 °C. The sarkosyl-insoluble pellet was taken up in 540 μl of Ca^2+^- and Mg^2+^-free PBS (pH 7.4) and used for stereotaxic injections. The concentration of alpha-synuclein in sarkosyl-insoluble fractions was measured with the Sensolyte anti-alpha-synuclein ELISA kit (Anaspec) according to the manufacturer’s directions. Briefly, sarkosyl insoluble fractions were diluted in the buffer provided and incubated in precoated wells overnight at 4 °C. The wells were washed seven times with the provided wash buffer. After a final wash, the color was developed using the TMB-ELISA substrate provided. Optical densities were measured at 450 nm with a Fluostar Omega microplate reader (BMG Labtech).

**Table 1:** Characteristics of MSA and iLBD cases used for inoculation experiments

Case ID	Sex (M/F)	Age at death (years)	Postmortem delay time (h)	Neurological diagnosis	Alpha-synuclein concentration in the sarkosyl-insoluble fraction (ng/ml)	Source
MSA1	F	51	48	MSA	20.99	BBCT
MSA2	M	59	32	MSA	21.52	QSBB
iLBD1	M	78	48	non-clinical	18.05	BBCM
iLBD2	F	68	48	non-clinical	8.17	BBCL

*MSA* multiple system atrophy, *iLBD* incidental Lewy body disease, *M* male, *F* female, *BBCT* Brain Banking Center Tübingen, *QSBB* Queen Square Brain Bank for Neurological Disorders, *BBCM* Brain Banking Center Münster, *BBCL* Brain Banking Center Leipzig

### Stereotaxic surgery

Six- to eight-week-old FVB;129S6-*Snca*^*tm1Nbm*^Tg(SNCA)1Nbm/J mice (short: Tg(SNCA)1Nbm/J mice, The Jackson Laboratory) were anaesthetized with isoflurane and stereotaxically injected with 30 μl of cortical brain extract from MSA or probable iLBD cases or PBS into the left striatum (coordinates: +0.2 mm relative to the bregma, +2.0 mm relative to the midline, 2.6 mm below the dura) using a 27-gauge disposable hypodermic syringe. After recovery from surgery, animals were monitored daily for health and three times weekly for signs of neurologic illness such as reduced grooming, ataxia, tremor, bradykinesia, akinesia, lethargy, circling, tail rigidity, paraparesis, paralysis, kyphosis, and more. At 3, 6, and 9 months post injection the mice were sacrificed by overdose with ketamine/xylazine and then transcardially perfused with 0.9 % saline followed by 10 % formalin neutral buffer solution (Sigma). Brains were fixed overnight with 1 % formalin neutral buffer solution for immunohistochemistry. For biochemical analysis, brains were snap-frozen on dry ice and stored at −80 °C. All animal studies were approved by the animal protection committee of the North Rhine-Westphalia State Environment Agency (LANUV).

### Immunohistochemical analysis

For paraffin sections brains fixed in 1 % formalin were dehydrated and infiltrated with paraffin using standard procedures. Brains were cut into 8-μm-thick coronal sections, mounted on glass slides, deparaffinized, and hydrated through a series of graded ethanol solutions followed by antigen-retrieval treatment for 10 min (Additional file 1). Endogenous tissue peroxide was inhibited by incubating slides in 3 % hydrogen peroxide solution for 30 min. Slides were then blocked with 30 % (v/v) normal goat serum, 1 % (v/v) bovine serum albumin, and 0.5 % Triton X-100 for 1 h and then incubated with primary antibody overnight at 4 °C (Additional file 1). Bound antibody was detected using a Vectastain ABC kit (Vector Laboratories) and visualized using DAB (3-3’-diaminobenzidine). Slides were counterstained with Hematoxylin QS (Vector Laboratories) and coverslipped with Vectamount AQ (Vector Laboratories). All slides were scanned using an Axio Scan.Z1 scanner and ZEN lite software (Carl Zeiss). For cryosections brains were fixed in 4 % formalin and protected in 30 % sucrose solution in PBS using standard procedures. Brains were cut into 25-μm-thick coronal sections, followed by antigen-retrieval treatment (Additional file 1). Staining was performed on free-floating sections. Brain sections were blocked with 10 % (v/v) normal goat serum and 0.3 % Triton X-100 for 1 h and incubated with primary antibody overnight as above. Bound antibody was detected using a Liquid DAB+ Substrate Chromagen System (Dako). The sections were mounted on slides, counterstained, coverslipped, and analyzed as above.

### Immunofluorescence analysis

Paraffin-embedded tissues were cut into 8-μm-thick coronal sections, deparaffinized, and hydrated followed by antigen retrieval treatment for 10 min (Additional file 1). The tissues were incubated in 1 mM CuSO_4_ in 50 mM ammonium acetate buffer at pH 5. Slides were blocked with 30 % (v/v) normal goat serum, 1 % (v/v) BSA, and 0.5 % Triton X-100 for 1 h and then incubated with primary antibody overnight at 4 °C (Additional file 1). After washing twice with PBS containing 0.25 % (v/v) Triton X-100 and once with PBS, sections were incubated with secondary antibodies conjugated to Alexa Fluor 488- or Alexa Fluor 568-conjugated goat anti-mouse or rabbit antibodies (Thermo Fisher Scientific). Sections were stained with DAPI (4’,6-diamidino-2-phenylindole; Thermo Fisher Scientific), coverslipped with Fluoromount (Sigma), and visualized using an LSM700 confocal microscope (Carl Zeiss).

### Biochemical analysis

Mouse brains were fractionated as described above for the preparation of human brain extracts for injections. However, the sarkosyl-soluble supernatant and the sarkosyl-insoluble pellet were taken up in Ca^2+^- and Mg^2+^-free PBS (pH 7.4) with protease and phosphatase inhibitors (cOmplete and PhosSTOP, Roche Diagnostics) and benzonase nuclease (Sigma) for the removal of nucleic acid. Protein concentrations were determined with the Pierce BCA Protein Assay Kit (Thermo Fisher Scientific). Samples were taken up in 1× NuPAGE loading buffer, boiled for 10 min, and then loaded onto 4-12 % NuPAGE gels (Thermo Fisher Scientific). Insoluble fractions of human brains were prepared as described above for injections. However, the sarkosyl-insoluble pellet was taken up in 500 μl of 30 mM Tris–HCl (pH 7.4) and sonicated. A mouse monoclonal anti-alpha-synuclein antibody (clone 42, BD Biosciences) was incubated at a 1:20 dilution with 50 μl of Dynabeads protein G slurry (Thermo Fisher Scientific) for 30 min at room temperature. Diluted insoluble fractions were incubated with the immune complex for 1 h at room temperature. The resultant pellet (P1) was washed three times with wash buffer and eluted with 20 μl of elution buffer and 10 μl of 4× loading buffer. The supernatant was subjected to a second immunoprecipitation step with a fresh immune complex overnight at 4 °C. The resultant pellet was washed three times with wash buffer. Finally, immunoprecipitated alpha-synuclein was eluted in 20 μl of elution buffer and 10 μl of 4× loading buffer, combined with the first pellet (P1), and boiled for 10 min. Protein was resolved on 4-12 % NuPAGE gels. SDS-PAGE (sodium dodecyl sulfate-polyacrylamide gel electrophoresis) was performed using a MOPS buffer system (Thermo Fisher Scientific), and gels were subsequently transferred to PVDF membranes using a wet blotting system. Blots were then cross-linked with 0.4 % (v/v) paraformaldehyde in PBS for 30 min. Membranes were blocked for 1 h at room temperature with 5 % (w/v) bovine serum albumin in Tris-buffered saline containing 0.05 % (v/v) Tween 20 and then incubated with primary antibody overnight at 4 °C (Additional file 1). Blots were developed with IRDye 680- or IRDye 800-conjugated goat anti-mouse or goat anti-rabbit secondary antibodies (LI-COR Biosciences), and imaged with an Odyssey infrared imaging system (LI-COR Biosciences).

Sequential extraction was performed as previously described [61]. Briefly, 10 % (w/v) brain homogenates prepared in Ca^2+^- and Mg^2+^-free PBS (pH 7.4) were shifted to high-salt (HS) buffer by the addition of NaCl to a final concentration of 750 mM and EDTA to a final concentration of 5 mM. One hundred microliters of brain homogenate in HS buffer were sonicated for 5 min using a water-bath sonicator and then centrifuged at 100,000 × *g* for 30 min at 4 °C. The pellet was then resuspended by pipetting in 100 μl of HS-T buffer [50 mM Tris–HCl (pH 7.5), 750 mM NaCl, 5 mM EDTA, 1 % (v/v) Triton X-100]. Samples were then sonicated and centrifuged at 100,000 × *g* for 30 min at 4 °C. Cycles of extraction, sonication, and centrifugation were repeated using the following buffers in sequence: RIPA buffer [50 mM Tris–HCl (pH 7.5), 150 mM NaCl, 5 mM EDTA, 1 % (v/v) Nonidet P-40, 0.5 % (w/v) sodium deoxycholate, 0.1 % (w/v) SDS] and SDS buffer [50 mM Tris–HCl (pH 7.5), 2 % (w/v) SDS]. Protease and phosphatase inhibitors were added to buffers before use. For each extraction step, a 60 μl sample of the supernatant was removed, adjusted to 1× NuPAGE loading buffer by the addition of 20 μl of 4× loading buffer and then boiled. Samples from each of the extraction steps were analyzed by immunoblotting as described before.

To determine alpha-synuclein expression in different brain regions of non-transgenic and Tg(SNCA)1Nbm/J mice, tissue from these regions was collected and homogenized in Ca^2+^- and Mg^2+^-free PBS (pH 7.4) to yield a 10 % (w/v) homogenate to which sarkosyl was added to yield a final concentration of 2 % (v/v) sarkosyl. Protein concentrations were determined using the Pierce BCA Protein Assay Kit, and samples of 5 μg total protein were separated on 4-12 % NuPAGE gels as described above.

### Production and purification of recombinant alpha-synuclein

Alpha-synuclein cDNA was transferred from plasmid pMA-T-SCNA (Thermo Fisher Scientific) as an NheI-XhoI fragment into the expression plasmid pET28a(+) (Merck) yielding pET28a(+)-aSWT. The *E. coli* strain BL21(DE3) (Merck) was transformed with sequence verified pET28a(+)-aSWT and individual clones were screened for production of recombinant protein following induction with isopropyl-D-thiogalactopyranoside (IPTG, Sigma). Expression with this system leads to translocation of the 6His-alpha synuclein wild-type protein into the periplasmatic space. For protein production, *E. coli* cells were grown at 37 °C in 1 L LB media containing ampicillin and 1 % glucose to an OD600 of approximately 0.5, induced with 1 mM IPTG and grown for 5 h at 37 °C. The periplasmatic material was released into buffer using an osmotic shock protocol [2]. Briefly, cells were pelleted by centrifugation at 6,000 × *g* for 15 min. The pellet was resuspended in a 35 % sucrose solution in 2 mM EDTA and 30 mM Tris–HCl (pH 7.2) and incubated with shaking at room temperature for 15 min. The cells were again harvested and resuspended in ice-cold water containing 5 mM MgSO_4_. The periplasmatic material was boiled for 20 min and then centrifuged for 30 min at 21,000 × *g*. 6His-alpha-synuclein was purified from the supernatant by Ni-NTA affinity chromatography using 20 mM sodium phosphate buffer, pH 7.4, 500 mM NaCl, and 10 mM imidazole as binding buffer and 20 mM sodium phosphate buffer (pH 7.4), 500 mM NaCl, and 500 mM imidazole as elution buffer on an ÄKTA pure chromatography system (GE Healthcare). The protein was dialyzed against 150 mM NaCl in 20 mM Tris–HCl (pH 7.2), and used for seeding assays.

### Thioflavin T binding assay

Kinetic seeding aggregation assays were performed by incubating his-tagged monomeric alpha-synuclein (50 μM) in low-binding black 96-well plates (Corning) at 37 °C in TBS with 20 μM Thioflavin T (Sigma) and 0.05 % (w/v) sodium azide (Sigma). Glass balls with 2 mm in diameter (Hecht) were distributed into each well of the 96-well plate to increase agitation. Seeds, 4 μl of a 10 % brain homogenate, were sonicated for 15 min in in a TK-52 water bath sonifier (Bandelin) before adding to the samples. Fluorescence (440 nm excitation, 485 nm emission, top read) was measured in an Envision multilabel reader (Perkin Elmer) at 15 min intervals preceded by 45 s shaking at 300 rpm in orbital mode with a 2 mm diameter.

## Results

**Fig. 1:**
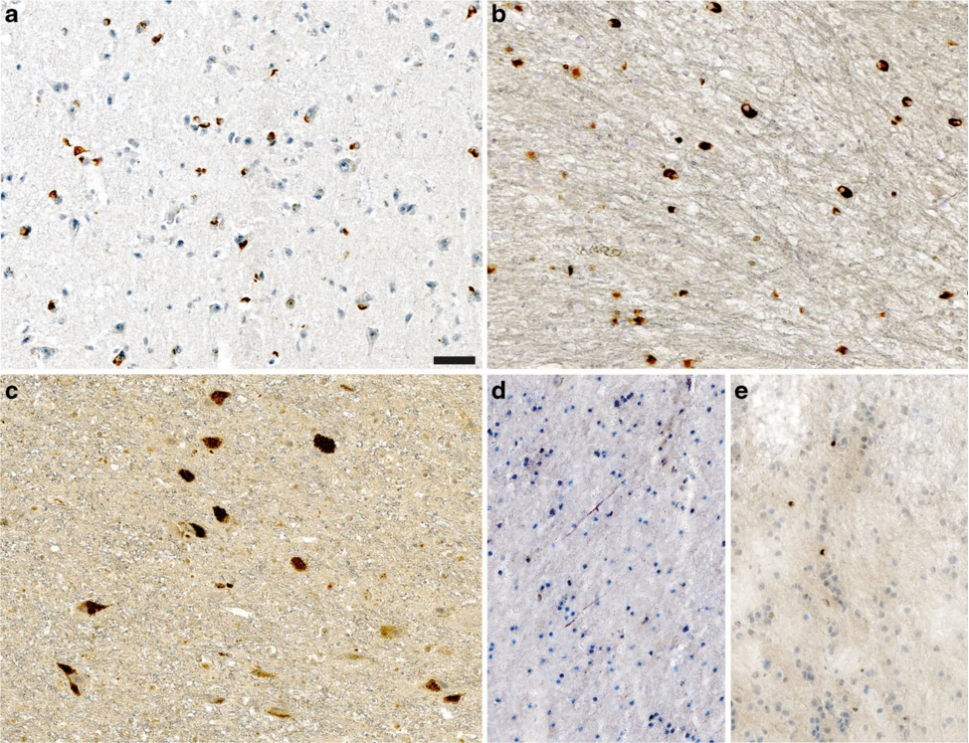
Brains of MSA and probable iLBD cases contain pathogenic alpha-synuclein. Immunohistochemical staining with an antibody against phosphorylated alpha-synuclein (81A) revealed glial cytoplasmic inclusions in cortical brain tissue sections from the MSA1 patient **a** and in cerebellar brain tissue sections from the MSA2 patient **b**. Staining of cortical brain tissue sections with the same antibody also showed granular deposits of phosphorylated alpha-synuclein in the cytoplasma and in neurites of cortical neurons from the iLBD1 **c** and iLBD2 cases **d**, **e**. Scale bar = 50 μm

**Fig. 2:**
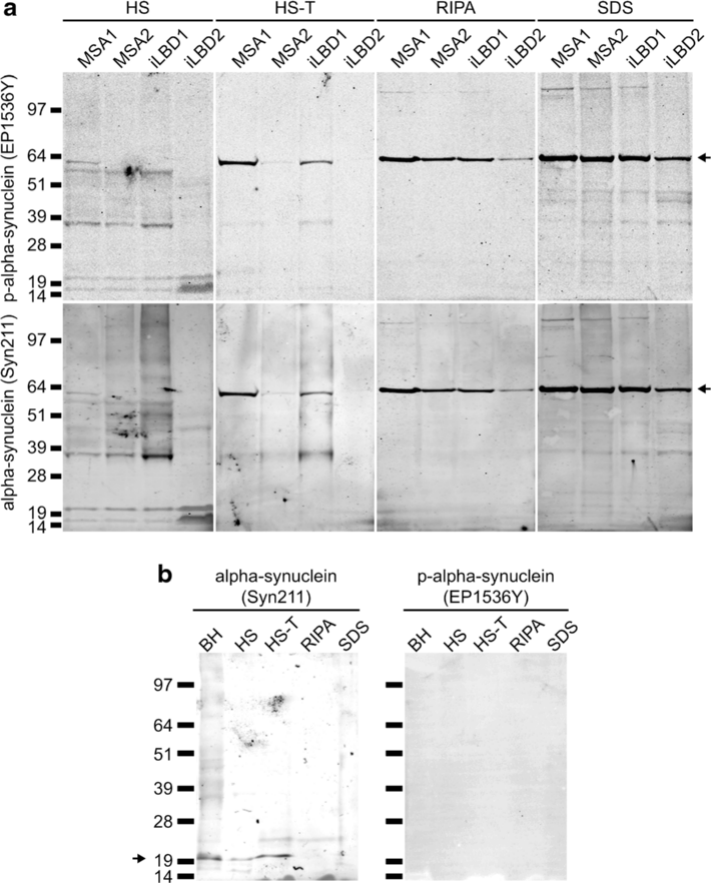
Biochemical analysis of alpha-synuclein in patient brains. Biochemical analysis of alpha-synuclein in patient brains shows that SDS-extractable phosphorylated alpha-synuclein was only observed in samples from patient brains **a** but not in a control brain **b**. Sequentially extracted fractions from brain homogenates of the MSA1, MSA2, iLBD1, and iLBD2 cases contain similar monomeric and oligomeric species of alpha-synuclein when probed with the EP1536Y antibody against phosphorylated alpha-synuclein (upper panel) and with the Syn211 antibody against alpha-synuclein (lower panel). Both antibodies recognize a prominent band at 64 kDa that may represent a tetramer (arrow) **a** and that is absent in control brain **b**. Sequential extractions from a control brain did not reveal phosphorylated alpha-synuclein and only showed monomeric alpha-synuclein in brain homogenate (BH), high salt (HS), and high salt-triton (HS-T) fractions (arrow) but not in the RIPA or SDS fraction **b**. Molecular sizes are shown in kilodalton

To investigate whether human wild-type alpha-synuclein supports prion-like spreading of pathological alpha-synuclein, we prepared and injected the sarkosyl-insoluble fraction from cortical brain tissue from two patients with MSA, a 51-year-old female and a 59-year-old male, and from two cases of probable iLBD, a 68-year-old female and a 78-year-old male (Table 1), without clinical symptoms of parkinsonism or any other neurological disorder, or phosphate-buffered saline (PBS) as a negative control into the striatum of 6-8-week-old adult Tg(SNCA)1Nbm/J mice (Table 2). Immunohistochemical analysis of brain tissue sections of the two MSA and two probable iLBD cases revealed that all patients displayed phosphorylated alpha-synuclein in their brains, albeit in different cell types and at different levels (Fig. 1a-e). Both MSA cases displayed prominent glial cytoplasmic inclusions that are characteristic for MSA (Fig. 1a and b), whereas the inclusions in both probable iLBD cases were neuronal as they are observed in PD or DLB (Fig. 1c-e). Biochemical analysis also revealed that these patient brains contained detergent-insoluble phosphorylated alpha-synuclein species that were absent in a control brain (Fig. 2 and Additional file 2). We also observed a prominent 64 kDa band that was observable in the RIPA and SDS fractions with the EP1536Y antibody against phosphorylated alpha-synuclein and the Syn211 antibody against alpha-synuclein.

**Table 2:** Summary of intrastriatal injections into Tg(SNCA)1Nbm/J mice

Number of mice injected	Injected material^a^	Time post-injection when the animal was euthanized (months)	Spread of pathology
4	30 μl PBS	3	No
4	30 μl PBS	6	No
4	30 μl PBS	9	No
4	30 μl MSA1 BE	3	No
4	30 μl MSA1 BE	6	Yes
4	30 μl MSA1 BE	9	Yes
4	30 μl MSA2 BE	3	No
4	30 μl MSA2 BE	6	Yes
4	30 μl MSA2 BE	9	Yes
4	30 μl iLBD1 BE	3	No
4	30 μl iLBD1 BE	6	Yes
4	30 μl iLBD1 BE	9	Yes
4	30 μl iLBD2 BE	3	No
4	30 μl iLBD2 BE	6	Yes
4	30 μl iLBD2 BE	9	Yes

*PBS* phosphate-buffered saline, *BE* brain extract, *MSA* multiple system atrophy, *iLBD* incidental Lewy body disease

**Fig. 3:**
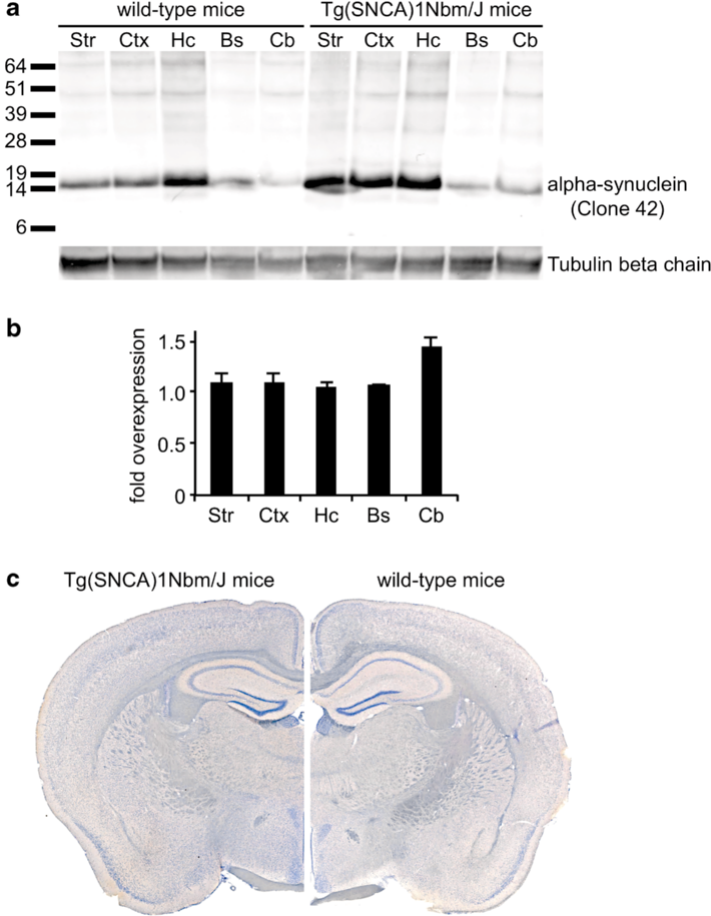
Biochemical and histological analysis of alpha-synuclein expression in wild-type and Tg(SNCA)1Nbm/J mice. Biochemical analysis with an antibody against alpha-synuclein (clone 42) that equally reacts with human as well as mouse alpha-synuclein shows that human alpha-synuclein is only very moderately overexpressed in Tg(SNCA)1Nbm/J mice in comparison to alpha-synuclein expression in wild-type mice. Equal amounts of total protein (5 μg) were loaded onto each lane of the SDS-polyacrylamide gel and show expression of alpha-synuclein in the striatum (Str), cortex (Ctx), hippocampus (Hc), brainstem (Bs), and cerebellum (Cb). Molecular sizes are shown in kilodalton **a**. The signal for alpha-synuclein was quantified by densitometry from western blots and normalized against tubulin and is shown as fold overexpression of alpha-synuclein in Tg(SNCA)1Nbm/J mice (n = 3) versus wild-type mice (n = 3) **b**. Immunohistochemistry of mouse brain sections with the same antibody (clone 42) against alpha-synuclein reveals comparable localization of alpha-synuclein in Tg(SNCA)1Nbm/J and wild-type mice **c**

Tg(SNCA)1Nbm/J mice are knockout for mouse alpha-synuclein and homozygously express human wild-type alpha-synuclein from an integrated P1 artificial chromosome that contains the entire human SNCA gene with its normal exon and intron structure as well as 35 kb of upstream sequences [14, 21]. Brain protein levels of human wild-type alpha-synuclein have been reported to be only modestly elevated in these mice (1.3-2.0-fold). Further analysis showed that the expression and localization of alpha-synuclein in the brain is comparable between wild-type and Tg(SNCA)1Nbm/J mice (Fig. 3). Moreover, these mice do not show overt alpha-synuclein aggregation in brain or colon or any enteric nervous system abnormalities. No detectable motor behavior impairments, autonomic abnormalities, olfactory dysfunction, dopaminergic deficits, Lewy body pathology, or neurodegeneration have been associated with human alpha-synuclein expression in these mice. We monitored these mice carefully for up to 9 months post injection. Within this period none of the injected mice developed visible signs of neurological disease.

**Fig. 4:**
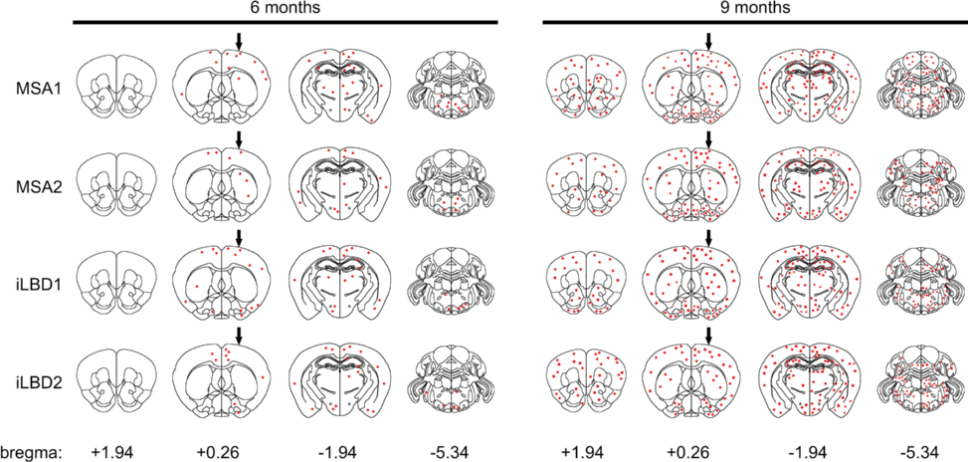
Prion-like spreading of phosphorylated alpha-synuclein. The diagram depicts areas in the brains of Tg(SNCA)1Nbm/J mice with inclusion bodies that stained positive for phosphorylated alpha-synuclein at 6 and 9 months after intrastriatal injection with brain extracts from MSA or probable iLBD cases into the left striatum (arrow). Staining for phosphorylated alpha-synuclein was verified with three different antibodies, 81A, EP1536Y, and pSyn#64. Mice injected with PBS were devoid of staining for phosphorylated alpha-synuclein and inclusion bodies for up to 9 months post injection. In mice injected with brain extracts from MSA or probable iLBD cases inclusion bodies became first visible at 6 months post injection and were more abundant in the left brain hemisphere where the injection had taken place. At 9 months post injection inclusion bodies with staining for phosphorylated alpha-synuclein were more abundant and detectable to near equal amounts in both hemispheres and had also spread to more rostral and caudal areas

**Fig. 5:**
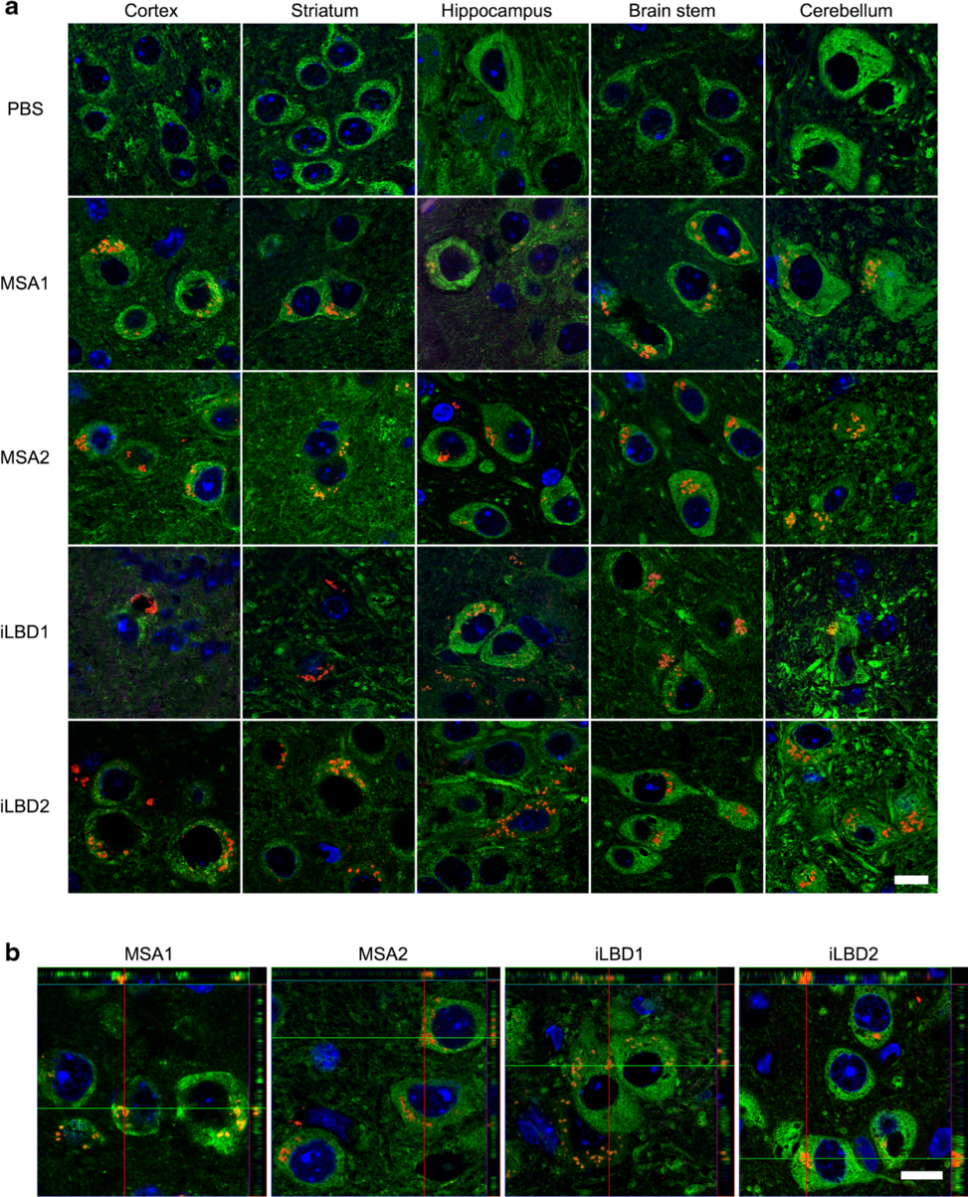
Accumulation of inclusion bodies in Tg(SNCA)1Nbm/J mice injected with brain extracts from MSA or probable iLBD cases. **a** Confocal images show immunofluorescence staining with the 81A antibody for phosphorylated (S129) alpha-synuclein (red) at 9 months post injection in brain sections of Tg(SNCA)1Nbm/J mice injected with brain extracts from MSA or probable iLBD cases or PBS. Neurons were stained with an antibody against tubulin beta-3 chain (green) and nuclei with DAPI (blue). Staining for phosphorylated alpha-synuclein was absent in mice injected with PBS. In contrast, mice injected with brain extracts from MSA or probable iLBD cases accumulated inclusion bodies with staining for phosphorylated alpha-synuclein in many areas of the brain, including the cortex, striatum, hippocampus, brain stem, and cerebellum. **b** Confocal images with orthogonal projections show that inclusion bodies with staining for phosphorylated alpha-synuclein (red) were located within neuronal cell bodies. Scale bars = 10 μm

We sacrificed Tg(SNCA)1Nbm/J mice at 3, 6, and 9 months post injection. Brain tissue sections of mice injected with PBS did not show any staining for phosphorylated alpha-synuclein for up to 9 months post injection (Fig. 5a). In contrast, brains of mice injected with cortical extracts from MSA or probable iLBD cases accumulated abnormal intraneuronal inclusion bodies with phosphorylated alpha-synuclein, which first became visible at 6 months post injection (Figs. 4 and 5). At this time point the pathological inclusion bodies containing phosphorylated alpha-synuclein were prominent in the ipsilateral brain hemisphere and only few aggregates were observed in the contralateral hemisphere (Fig. 4). No phosphorylated alpha-synuclein had accumulated in rostral areas and only very little in caudal areas. At 9 months post injection aggregates with phosphorylated alpha-synuclein were more abundant, equally present in both brain hemispheres, and had spread to more rostral and caudal areas in the brain (Figs. 4 and 5, and Additional file 3). The spatiotemporal spread of synucleinopathy in the CNS was comparable between all animals injected with MSA and iLBD brain extracts for the observed time points (Table 2). Confocal analysis showed that aggregates with phosphorylated alpha-synuclein were indeed confined to neuronal cell bodies (Fig. 5b). Only occasionally did phosphorylated alpha-synuclein aggregates localize to astrocytes or microglia (Additional file 4).

**Fig. 6:**
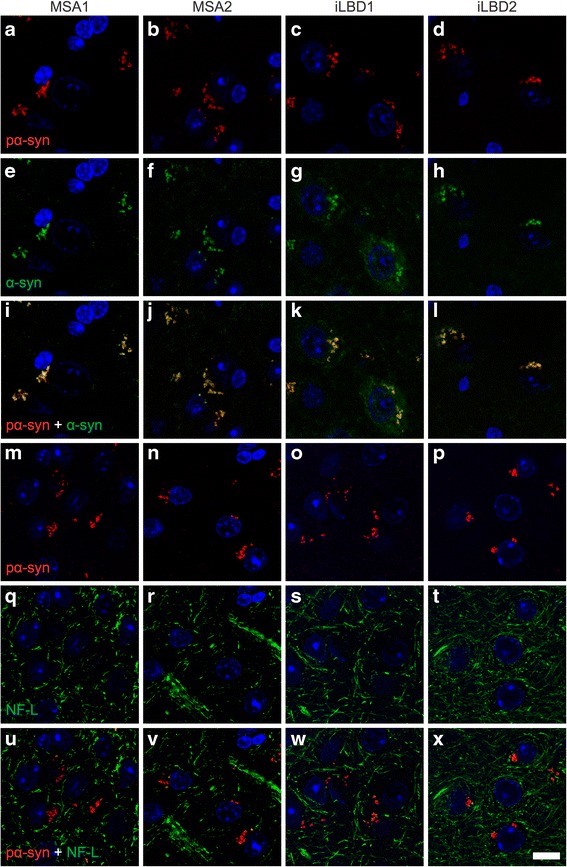
Staining for phosphorylated alpha-synuclein is specific. Confocal imaging of brain sections of Tg(SNCA)1Nbm/J mice injected with brain extracts from MSA or probable iLBD cases shows that at 9 months post injection staining with the EP1536Y antibody for phosphorylated (S129) alpha-synuclein **a**-**d** and staining with the Syn211 antibody for human alpha-synuclein **e**-**h** colocalize when merged **i**-**l**. In contrast, staining with the 81A antibody for phosphorylated alpha-synuclein **m**-**p** does not colocalize with staining for neurofilament light polypeptide **q**-**t** when merged **u**-**x**. Nuclei were stained with DAPI (blue). Scale bar = 10 μm

Staining for phosphorylated alpha-synuclein colocalized with staining for alpha-synuclein only (Fig. 6a-l), indicating that this antibody against phosphorylated alpha-synuclein is targeting an epitope specific for a subpopulation of alpha-synuclein. While the 81A antibody against phosphorylated alpha-synuclein may also nonspecifically recognize neurofilament light polypeptide (also known as NF-L) [48], here staining for neurofilament light polypeptide did not colocalize with that for phosphorylated alpha-synuclein (Fig. 6m-x).

**Fig. 7:**
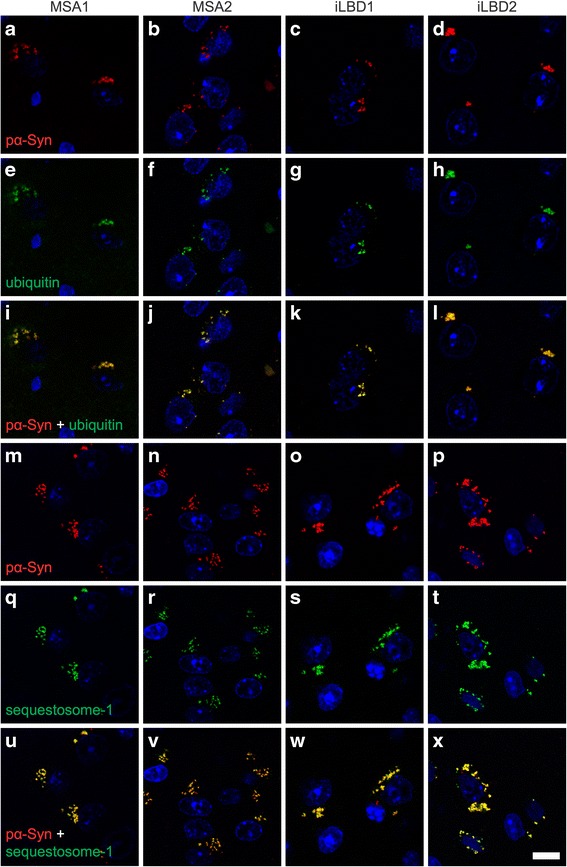
Phosphorylated alpha-synuclein in inclusion bodies colocalizes with ubiquitin and sequestosome-1. Confocal imaging of brain sections of Tg(SNCA)1Nbm/J mice injected with brain extracts from MSA or probable iLBD cases shows that at 9 months post injection staining with the EP1536Y antibody for phosphorylated alpha-synuclein **a**-**d** and staining for ubiquitin **e**-**h** colocalize when merged **i**-**l**. In addition, staining with the 81A antibody for phosphorylated alpha-synuclein **m**-**p** colocalizes with staining for sequestosome-1 **q**-**t** when merged **u**-**x**. Nuclei were stained with DAPI (blue). Scale bar = 10 μm

Further analysis of the intraneuronal inclusion bodies with phosphorylated alpha-synuclein showed that these aggregates additionally contained several proteins involved in protein degradation. Staining for phosphorylated alpha-synuclein colocalized with that for ubiquitin (Fig. 7a-l). Ubiquitin often labels pathological inclusions that appear to be resistant to normal degradation including alpha-synuclein in Lewy bodies and in glial and neuronal cytoplasmic inclusions in MSA [23, 26, 52, 53]. In addition, staining for phosphorylated alpha-synuclein also colocalized with that for sequestosome-1 (also known as p62) (Fig. 7m-x and Additional file 5), which binds to polyubiquitinated protein aggregates such as alpha-synuclein in Lewy bodies [22]. Occasionally, few smaller sized aggregates of sequestosome-1 could be detected that did not colocalize with phosphorylated alpha-synuclein (Additional file 5).

Next, we investigated whether Tg(SNCA)1Nbm/J mice injected with cortical extracts from MSA or probable iLBD cases or PBS had developed any form of gliosis in their CNS at 9 months post injection. Staining with antibodies against glial fibrillary acidic protein (GFAP) revealed exclusively a mild reactive astrocytic gliosis in the hippocampus of all mice but nowhere else in the CNS, regardless if they had been injected with cortical extracts from MSA or iLBD cases or PBS (Additional file 6). This suggests that this transgenic mouse line has an intrinsic property to develop mild hippocampal astrocytic gliosis with age. Staining with antibodies against allograft inflammatory factor 1 (also known as Iba1) did not reveal augmented activation of microglia in mice injected with human brain extracts in comparison to mice injected only with PBS (Additional file 7). Thus, the injections did not cause any enhanced gliosis in Tg(SNCA)1Nbm/J mice at 9 months post injection.

**Fig. 8:**
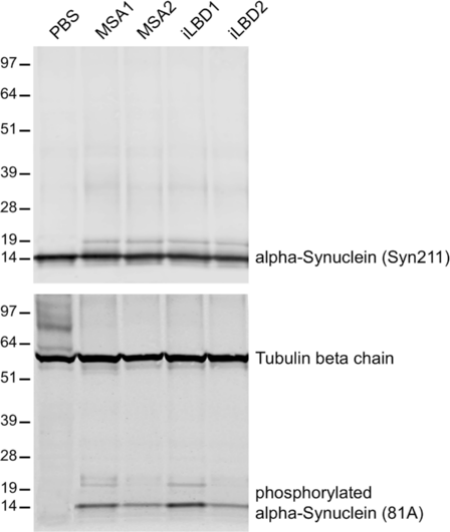
Brains of mice injected with brain extracts from MSA and probable iLBD cases but not PBS accumulate phosphorylated alpha-synuclein. Brains of Tg(SNCA)1Nbm/J mice injected with brain extracts from MSA or probable iLBD cases or PBS were biochemically analyzed at 9 months post injection with antibodies against alpha-synuclein (Syn211) or phosphorylated alpha-synuclein (81A). All mouse brains contained equal amounts of sarkosyl-soluble alpha-synuclein (upper panel). Phosphorylated alpha-synuclein, however, was not detectable in mouse brains injected with PBS but in mouse brains injected with brain extracts from MSA or probable iLBD cases (lower panel). Detection of tubulin beta chain shows equal loading in all lanes. Molecular sizes are shown in kilodalton

Consistent with the confocal imaging results, the biochemical analysis showed phosphorylated alpha-synuclein in the brains of mice injected with cortical extracts from MSA and probable iLBD cases but not in mice injected with PBS at 9 months post injection (Fig. 8). None of the injected mouse brains contained detectable levels of phosphorylated alpha-synuclein species that were insoluble in sarkosyl (Additional file 8) indicating that the intraneuronal inclusion bodies, which had accumulated over time, were not Lewy body-like but instead represented an early stage synucleinopathy that might precede Lewy body pathology.

**Fig. 9:**
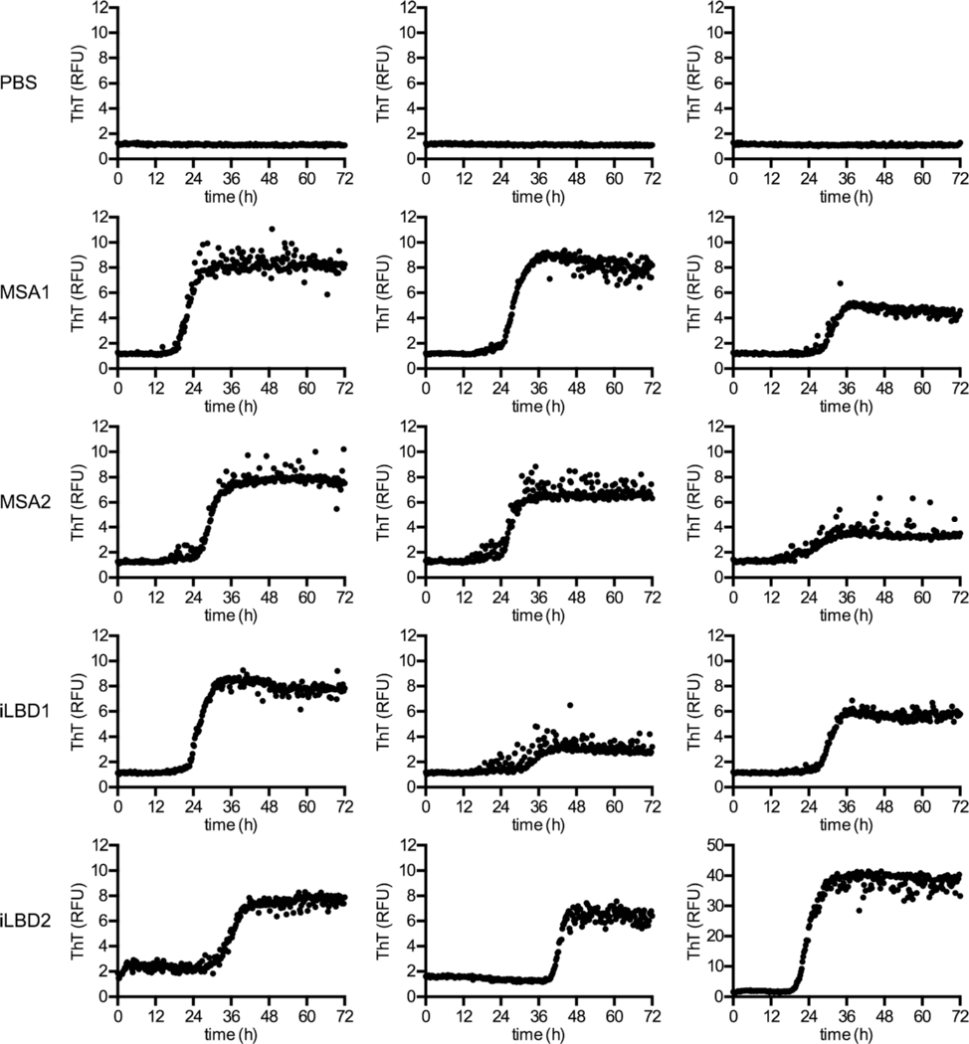
Thioflavin T binding assay detects abnormal alpha-synuclein in the brains of mice injected with brain extracts from MSA and iLBD patients. Shown is the time dependent increase in Thioflavin T (ThT) fluorescence measured in relative fluorescence units (RFU) over a period of 72 h. Brain homogenates from mice injected with PBS did not seed aggregation of alpha-synuclein, suggesting that they did not contain abnormal alpha-synuclein. In contrast, brain homogenates from mice injected with brain extracts from patients with MSA1, MSA2, iLBD1, and iLBD2 lead to an increase in ThT fluorescence over time, suggesting that the brains of these mice contained abnormal alpha-synuclein that was seeding competent. Each row represents replicates from the same whole brain homogenate of mice collected at 9 months post injection

To better identify the abnormal conformational change of alpha-synuclein in the brains of mice injected with brain extracts from patients with MSA and iLBD we performed Thioflavin T (ThT) binding assays (Fig. 9). Our results show that in contrast to brain homogenates from mice injected with PBS those injected with brain extracts from MSA and iLBD cases seeded aggregation of recombinant monomeric alpha-synuclein. This data further supports our histological findings that brain extracts from MSA and iLBD cases are able to induce templated misfolding and prion-like propagation of alpha-synuclein in Tg(SNCA)Nmb1/J mice.

## Discussion

In this study we have shown that intrastriatal injection of cortical brain extracts from MSA and iLBD cases into mice expressing human wild-type alpha-synuclein but not endogenous mouse alpha-synuclein causes the appearance and prion-like spreading of inclusion bodies that contain phosphorylated alpha-synuclein in vivo. Prion-like spreading of alpha-synuclein containing aggregates has been shown in M83-mice overexpressing human alpha-synuclein with the A53T-mutation and in wild-type mice after injection with synthetic alpha-synuclein fibrils made from recombinant protein [28, 29, 31]. Accumulation of phosphorylated alpha-synuclein has also been shown after inoculation of alpha-synuclein fibrils in M20-mice overexpressing human alpha-synuclein in the presence of endogenously expressed mouse alpha-synuclein [12, 46]. Recently, one study showed that brain homogenates from MSA patients caused disease with incubation times of 143 ± 16 and 106 ± 11 days when injected in M83^+/−^-mice [61]. However, injection of wild-type mice with brain extracts from patients with dementia with Lewy bodies only led to deposits of alpha-synuclein in 50 % of the injected mice at 15 months post injection that was mostly restricted to the injected hemisphere [31]. Although human and mouse alpha-synuclein can cross-seed each other, it is possible that this inefficient transmission was due to a species barrier as it is often observed in prion transmission studies where differences in the primary structure between the endogenously expressed prion protein and that of the infectious prion protein in the inoculum can hamper cross-species transmission [50]. Moreover, prion transmission studies to transgenic mice expressing the human prion protein have shown that mice only became susceptible to infection with human prions from Creutzfeldt-Jakob patients when expression of the mouse prion protein was ablated and only the human prion protein was expressed [56, 57]. In this study, all Tg(SNCA)1Nbm/J mice injected with patient brain extracts developed synucleinopathy that efficiently spread to the contralateral hemisphere within 9 months post injection suggesting that sequence identity between the alpha-synuclein seeds from human brain extracts and human wild-type alpha-synuclein expressed in these mice and possibly also the lack of endogenously expressed mouse alpha-synuclein facilitated the prion-like spread. Interestingly, the lack of endogenously expressed mouse alpha-synuclein did not affect incubation times in M83^+/−^-mice after inoculation with MSA brain homogenates [39]. Our qualitative analysis showed widespread localization of phosphorylated alpha-synuclein in the CNS, including the cortex, of Tg(SNCA)1Nbm/J mice after injection with patient brain extracts. In spontaneously diseased M83-mice, there is some accumulation of phosphorylated-alpha-synuclein in the cortex but more in the cerebellum, brain stem, especially the mesencephalon, and spinal cord [12, 29]. The distribution of alpha-synuclein pathology is however different in M83-, M20-, and wild-type mice that have been injected with recombinant alpha-synuclein fibrils and in these cases can more strongly involve the cortex [28, 48]. Differences in the localization of phosphorylated alpha-synuclein may among other reasons arise from the type of material injected, in our case patient brain extracts versus recombinant fibrils of alpha-synuclein in the other mouse models. While it is still a matter of investigation whether different strains of alpha-synuclein exist [36, 39], in prion diseases, the pathology observed is dependent on the expression profile of the prion protein and the type of prion strain inoculated [5]. In analogy, differences in the expression profile of alpha-synuclein could also affect the accumulation pattern of alpha-synuclein pathology. Notably, alpha-synuclein expression is driven in Tg(SNCA)1Nbm/J mice from the human *SNCA* promoter and in M83- and M20-mice from the hamster *Prnp* promoter.

Interestingly, punctate aggregates of phosphorylated alpha-synuclein in the brains of Tg(SNCA)1Nbm/J mice injected with brains extracts from patients with MSA or iLBD colocalized with ubiquitin and p62 as has been observed in earlier transmission studies to other mouse models of synucleinopathies [31, 45–48]. In contrast to another study in which wild-type mice were injected with synthetic alpha-synuclein fibrils and accumulated dot-like inclusions of phosphorylated microtubule-associated protein tau and TAR DNA-binding protein-43 (also known as TDP-43), we did not observe accumulation of these proteins in the brains of Tg(SNCA)1Nbm/J mice at 9 months post injection of human brain extracts, which may reflect differences of injected material [30].

In contrast to the mouse models described above where alpha-synuclein pathology was first observed between 2 and 4 months post injection based on the model and alpha-synuclein deposits were insoluble in detergent, we detected alpha-synuclein pathology only at 6 months post injection in our animal model. Moreover, in our model the phosphorylated alpha-synuclein component in the inclusion bodies was soluble in sarkosyl suggesting that the inclusion bodies in injected Tg(SNCA)1Nbm/J mice represent an early state of synucleinopathy. We chose to terminate these experiments at 9 months post injection and it is possible that a longer incubation time after injection of MSA or iLBD brain extracts may have resulted in more severe pathology with sarkosyl-insoluble alpha-synuclein aggregates and clinical signs as observed in M83^+/−^-mice injected with brain homogenate from MSA patients [61] or wild-type mice injected with synthetic alpha-synuclein fibrils [28, 31]. A recent study also shows that intracerebral injections of 30 μl of a 1 % brain homogenate from two MSA patients did not cause visible signs of disease in Tg(SNCA)1Nbm/J mice within a year [39]. Alternatively, expression levels of wild-type human alpha-synuclein may be too low in Tg(SNCA)1Nbm/J mice to allow for a more severe type of synucleinopathy to develop. Cortical protein levels of the A53T-mutant of human alpha-synuclein in M83^+/−^-mice are 3.3 ± 0.5-fold and that of M20^+/−^-mice 5.6 ± 0.7-fold above that of mouse alpha-synuclein expressed in wild-type mice [12]. Moreover, M83^+/−^- and M20^+/−^-mice still express endogenous mouse alpha-synuclein, which could contribute to the changes observed in those mice considering that prion-like propagation of alpha-synuclein aggregates can be induced in wild-type mice [28, 31]. Also, human wild-type alpha-synuclein has a much lower propensity to aggregate than human alpha-synuclein carrying the A53T mutation, which is associated with familial PD, or than murine wild-type alpha-synuclein, which naturally carries threonine at this position [37, 44]. While this paper was in preparation another study reported induction of alpha-synuclein pathology in the brains of wild-type mice and macaques after injection with Lewy body extracts from brains of patients with PD [40]. Similar to our observations Lewy body-injected mice and monkeys did not appear to accumulate typical Lewy body-type aggregates but instead showed phosphorylated alpha-synuclein that adopted a more punctate, inclusion-like conformation. Importantly, unlike brain homogenates from mice injected with PBS, brain homogenates from mice injected with MSA and iLBD patient brain extracts were able to seed alpha-synuclein aggregation in vitro as determined by ThT binding assays, suggesting that these mouse brains contained abnormal misfolded alpha-synuclein that was seeding competent. Variation in the ThT fluorescence signal between the different samples may be attributable to stochastic sampling effects or possibly to a heterogeneous population of abnormal alpha-synuclein species in the homogenates. A limitation of ThT binding assay may be that it could detect residual seeds originating form the injected patient material. Considering that the histology results are negative for phosphorylated alpha-synuclein at 3 months post injection and only become positive at 6 months post injection for animals injected with patient brain extracts, this assay likely detects de novo generated seeds.

In contrast to PD and DLB, where aggregates of alpha-synuclein are mostly found in neurons, inclusions of misfolded alpha-synuclein in MSA have been mostly associated with oligodendrocytes in the past. A recent study however shows that in addition to glial inclusions also neuronal inclusions of alpha-synuclein are widespread and play an important role in the development and progression of MSA [6]. Interestingly, another study reported that the structural conformation (strain) of the injected alpha-synuclein aggregates, in this case fibrils versus ribbons, was found to induce alpha-synuclein inclusions only in neurons, as observed in PD, or in neurons and oligodendrocytes, as observed for MSA [36]. The latter, however, was observed only after combined rAAV-mediated alpha-synuclein overexpression with alpha-synuclein ribbons inoculation, which led to a second but sparse alpha-synuclein phosphorylation in oligodendrocytes.

In this study, brain extracts of MSA patients caused accumulation of phosphorylated alpha-synuclein mostly in neurons and rarely in oligodendrocytes of Tg(SNCA)1Nbm/J mice. With regard to observations in M20-mice injected with alpha-synuclein fibrils, we detected phosphorylated alpha-synuclein aggregates only occasionally in astrocytes and microglia (Additional file 4) [46], which may explain the lack of wide spread gliosis in our model system (Additional files 6 and 7). A lack of oligodendroglial accumulation of phosphorylated alpha-synuclein has also been observed in M83^+/−^-mice injected with brain homogenates from MSA patients and it was speculated that the lack of alpha-synuclein aggregation in oligodendrocytes might be due to poor transgene expression in these cells [39, 61]. Low expression levels of alpha-synuclein in oligodendrocytes may not support prion-like seeding and accumulation of alpha-synuclein after transfer from neurons [43]. Expression of alpha-synuclein in M20- and M83-mice are driven from the prion promoter, while expression of human alpha-synuclein in Tg(SNCA)1Nbm/J mice is driven from a P1 artificial chromosome, which contains the human promoter for alpha-synuclein. Although human oligodendrocytes were long thought not to express alpha-synuclein, a recent study revealed that the expression of alpha-synuclein in MSA neurons and glial cells is enhanced [1, 32]. Thus, the lack of alpha-synuclein pathology in oligodendrocytes of Tg(SNCA)1Nbm/J mice might be due to missing enhancer elements in the P1 artificial chromosome or to the lack of a yet unresolved mechanism in trans, governing the expression of alpha-synuclein in MSA. Our data does not argue for or against the existence of strains with regard to misfolded alpha-synuclein species and emphasize the necessity for additional studies to understand this matter in greater detail.

ILBD is a prodromal form of synucleinopathy, e.g. PD or DLB, that has not resulted in dementia or parkinsonism yet, and is found in 8-17 % of neurologically asymptomatic individuals over the age of 60 on post-mortem examination [7, 9, 10, 13, 16]. Cortical brain extracts from the two probable iLBD cases investigated here caused a similar spatiotemporally progressive synucleinopathy in Tg(SNCA)1Nbm/J mice as those from the two MSA patients, which lets us conclude that they were indeed true iLBD cases. Moreover, several other transmission studies to animal models in the past have shown that a positive transmission result is not to be expected in the absence of alpha-synuclein aggregates in the injected material as it is observed after injection with PBS [28, 29, 31, 47, 61]. The concentration of alpha-synuclein in the sarkosyl-insoluble fraction of the iLBD2 sample was slightly lower than in the other brain extracts (Table 1). Titration experiments in prion transmission studies show that only large differences in titers that can span several tenfold dilutions lead to large differences in the incubation time in inoculated animals [38, 54, 55]. We injected Tg(SNCA)Nbm1/J mice with a high dose of misfolded alpha-synuclein species from the sarkosyl-insoluble fraction of MSA and iLBD brains and did not observe any difference in the spatiotemporal expansion of alpha-synuclein pathology in the brains of injected mice. Only more comprehensive titration studies in mice with sarkosyl-insoluble fractions from brain extracts of patients with synucleinopathies will show at which concentrations of alpha-synuclein differences in the spread of this pathology becomes observable. Our findings argue that the pathologic alpha-synuclein species in the brains of patients with iLBD are comparable to those from MSA brains in their ability to induce pathology in Tg(SNCA)1Nbm/J mice.

## Conclusions

We show that human wild-type alpha-synuclein by itself, in the absence of endogenously expressed mouse alpha-synuclein, supports a prion-like mechanism in the spreading of pathological alpha-synuclein, which does not necessitate the presence of fulminant Lewy body-type pathology. In our experiments sarkosyl-soluble pathogenic alpha-synuclein species, which need to be further characterized, were readily transmitted between neurons supporting the concept that the appearance of additional detergent-insoluble alpha-synuclein species and Lewy body pathology may represent a late cellular event in mature synucleinopathies [19, 34, 41]. Consequentially, potential therapeutic strategies targeting the interneuronal spread of soluble pathogenic alpha-synuclein in synucleinopathies such as PD or MSA may be promising but effective only early in the disease process.

### Ethical approval

All applicable international, national, and/or institutional guidelines for the care and use of animals were followed.

## Additional files

Additional file 1: Table S1. Antibodies used for immunofluorescence, immunohistochemistry, and western blotting. (PDF 48 kb) Additional file 2:
**Brains of MSA and probable iLBD cases contain phosphorylated alpha-synuclein in the sarkosyl-insoluble fraction.** (PDF 1486 kb) Additional file 3:
**Accumulation of inclusion bodies in Tg(SNCA)1Nbm/J mice injected with brain extracts from MSA or probable iLBD cases.** (PDF 30979 kb) Additional file 4:
**Phosphorylated alpha-synuclein in inclusion bodies occasionally colocalizes with astrocytes and microglia.** (PDF 13484 kb) Additional file 5:
**Phosphorylated alpha-synuclein in inclusion bodies colocalizes with sequestosome-1/p62.** (PDF 5612 kb) Additional file 6:
**Reactive astrocytic gliosis in aged Tg(SNCA)1Nbm/J mice.** (PDF 13304 kb) Additional file 7:
**DAB-staining for Iba1 in aged Tg(SNCA)1Nbm/J mice.** (PDF 14000 kb) Additional file 8:
**Brains of mice injected with brain extracts from MSA and probable iLBD cases did not accumulate phosphorylated alpha-synuclein that was sarkosyl insoluble.** (PDF 398 kb)
