# Supplementary material for: Prion-like propagation of human brain-derived alpha-synuclein in transgenic mice expressing human wild-type alpha-synuclein
Source: Acta Neuropathol Commun. 2015 Nov 26;3:75. doi: 10.1186/s40478-015-0254-7 (PMC4660655; doi:10.1186/s40478-015-0254-7)
Supplement: Additional file 1: Table S1. — Antibodies used for immunofluorescence, immunohistochemistry, and western blotting. (PDF 48 kb) [file 40478_2015_254_MOESM1_ESM.pdf]

Table S1 Antibodies used for immunofluorescence, immunohistochemistry, and western blotting

Target (alternative name) [antibody clone]	Source	Catalogue number	Host	Dilution (IF/IHC)	Antigen retrieval[a]	Dilution (WB)
alpha-Synuclein [Syn211]	Merck Millipore	36-008	Mouse	1:100	Citrate buffer	1:2000
alpha-Synuclein [Clone 42]	BD Biosciences	610787	Mouse	1:500	Citrate buffer	1:1000
alpha-Synuclein (phospho S129) [81A]	Covance	MMS-5091	Mouse	1:200	Citrate buffer	1:1000
alpha-Synuclein (phospho S129) [EP1536Y]	Abcam	AB51253	Rabbit	1:200	Citrate buffer	1:1000
alpha-Synuclein (phospho S129) [pSyn#64]	Wako	015-25191	Mouse	–	Citrate buffer	1:1000
Glial fibrillary acidic protein (GFAP)	Dako	Z0334	Rabbit	1:400	Citrate buffer	–
Heat shock cognate 71 kDa protein (Hsc70)	Abcam	AB19136	Rat	1:100	Citrate buffer	–
Iba1	Wako	019-19741	Rabbit	1:400	Citrate buffer	–
Neurofilament light polypeptide (NF-L)	Cell Signalling	2837	Rabbit	1:200	Citrate buffer	1:1000
Tau (phospho-PHF-tau pSer202+Thr205) [AT8]	Thermo Scientific	MN1020B	Mouse	1:1000	Formic acid	–
TAR DNA-binding protein 43 (phospho TDP-43 pS409/410)	Cosmo Bio	TTP-PTD-P02	Rabbit	1:2000	Formic acid	–
Sequestosome-1 (p62)	Proteintech	18420-1-AP	Rabbit	1:100	Citrate buffer	–
Tubulin beta chain	Thermo Scientific	MA5-16308	Mouse	–	Citrate buffer	1:1000
Tubulin beta-3 chain	Covance	PRB-435P	Rabbit	1:1000	Citrate buffer	–
Ubiquitin (Ubi-1)	Merck Millipore	MAB1510	Mouse	1:500	Formic acid	–

a Formic acid treatment: 10% formic acid for 15 min at room temperature
