# Supplementary material for: Prion-like propagation of human brain-derived alpha-synuclein in transgenic mice expressing human wild-type alpha-synuclein
Source: Acta Neuropathol Commun. 2015 Nov 26;3:75. doi: 10.1186/s40478-015-0254-7 (PMC4660655; doi:10.1186/s40478-015-0254-7)
Supplement: Additional file 2: — Brains of MSA and probable iLBD cases contain phosphorylated alpha-synuclein in the sarkosyl-insoluble fraction. (PDF 1486 kb) [file 40478_2015_254_MOESM2_ESM.pdf]

Additional file 2 Brains of MSA and probable iLBD cases contain phosphorylated alpha-synuclein in the sarkosyl-insoluble fraction

The amount of alpha-synuclein in the sarkosyl-insoluble fraction of brain homogenate from the MSA1, MSA2, and iLBD1 cases was quantified (Table 1). Equal amounts of protein were used to immunoprecipitate alpha-synuclein from each sarkosyl-insoluble fraction with the clone 42 antibody against alpha-synuclein. Proteins were separated by SDS-polyacrylamide gel electrophoresis and detected with the pSyn#64 antibody against phosphorylated alpha-synuclein **(a)**. Phosphorylated alpha-synuclein was not detectable when the primary pSyn#64 antibody against phosphorylated alpha-synuclein was omitted **(b)**. * = IgG light chain. Molecular sizes are shown in kilodalton.

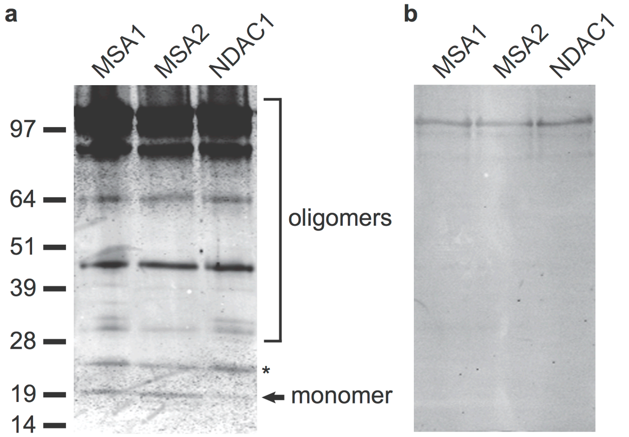
a
MSA1
MSA2
NDAC1
97
64
51
39
28
19
14
oligomers
*
monomer
b
MSA1
MSA2
NDAC1
