# Supplementary material for: Prion-like propagation of human brain-derived alpha-synuclein in transgenic mice expressing human wild-type alpha-synuclein
Source: Acta Neuropathol Commun. 2015 Nov 26;3:75. doi: 10.1186/s40478-015-0254-7 (PMC4660655; doi:10.1186/s40478-015-0254-7)
Supplement: Additional file 3: — Accumulation of inclusion bodies in Tg(SNCA)1Nbm/J mice injected with brain extracts from MSA or probable iLBD cases. (PDF 30979 kb) [file 40478_2015_254_MOESM3_ESM.pdf]

Additional file 3 Accumulation of inclusion bodies in Tg(SNCA)1Nbm/J mice injected with brain extracts from MSA or probable iLBD cases

DAB-staining with the pSyn#64 antibody for phosphorylated alpha-synuclein in brain sections of Tg(SNCA)1Nbm/J mice injected with brain extracts from MSA or probable iLBD cases shows at 9 months post injection punctate aggregates of phosphorylated alpha-synuclein (arrow heads). Brain sections from mice injected with PBS did not show any staining for phosphorylated alpha-synuclein. Sections were counterstained with hematoxylin. Scale bar = 10 µm.

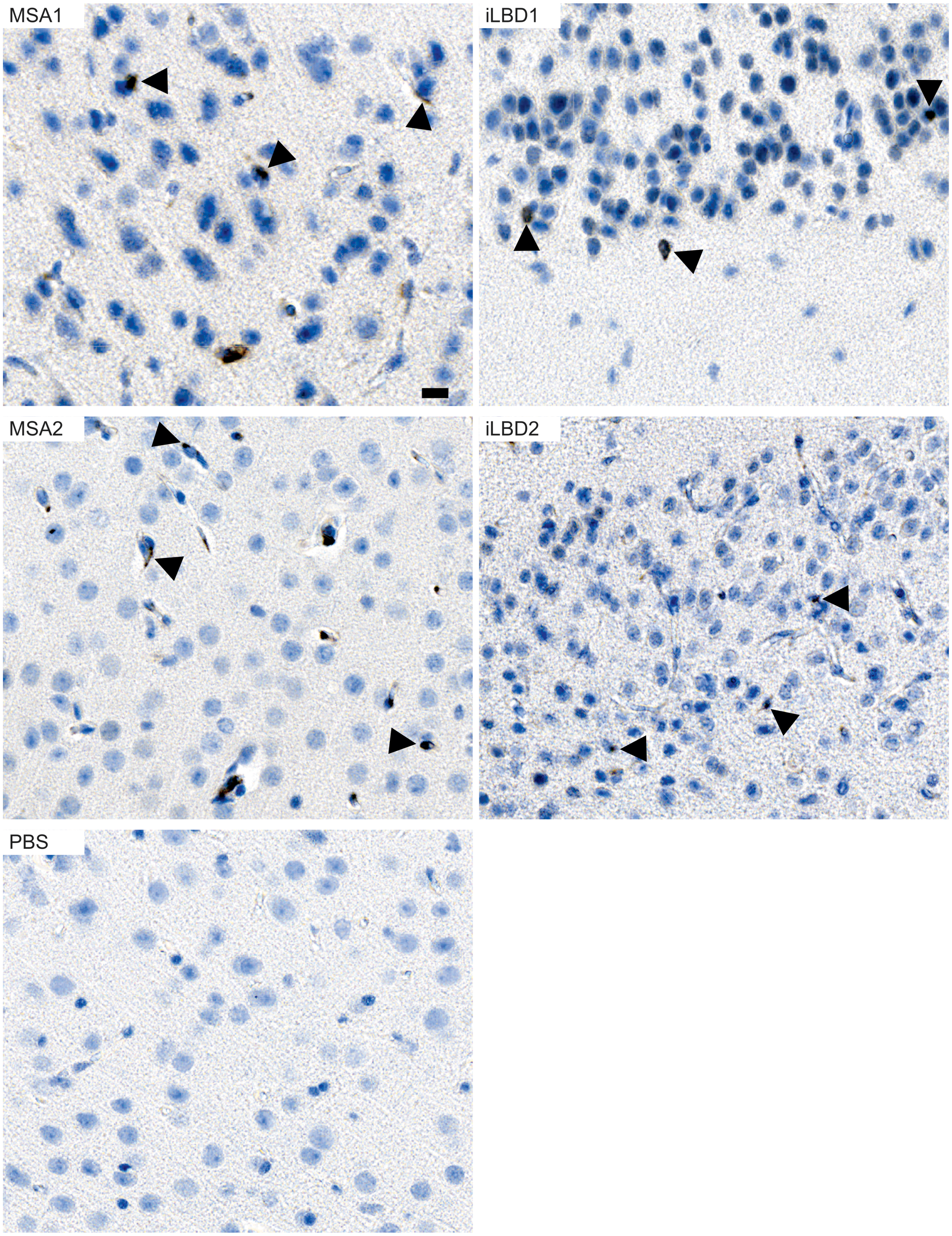
MSA1
iLBD1
MSA2
iLBD2
PBS
