# Supplementary material for: Prion-like propagation of human brain-derived alpha-synuclein in transgenic mice expressing human wild-type alpha-synuclein
Source: Acta Neuropathol Commun. 2015 Nov 26;3:75. doi: 10.1186/s40478-015-0254-7 (PMC4660655; doi:10.1186/s40478-015-0254-7)
Supplement: Additional file 4: — Phosphorylated alpha-synuclein in inclusion bodies occasionally colocalizes with astrocytes and microglia. (PDF 13484 kb) [file 40478_2015_254_MOESM4_ESM.pdf]

Additional file 4 Phosphorylated alpha-synuclein in inclusion bodies occasionally colocalizes with astrocytes and microglia

Confocal imaging of brain sections of Tg(SNCA)1Nbm/J mice injected with brain extracts from MSA or probable iLBD cases shows that at 9 months post injection punctate aggregates of phosphorylated alpha-synuclein, as seen with staining with the 81A antibody (**a**-**d**), occasionally localize to astrocytes, as seen with staining for GFAP (**e**-**h**), when both images are merged (**i**-**l**). In addition, staining with the 81A antibody for phosphorylated alpha-synuclein (**m**-**p**) shows that punctate aggregates of phosphorylated alpha-synuclein also occasionally localize to microglia, as seen with staining for Iba1 (**q**-**t**), when both images are merged (**u**-**x**). Nuclei were stained with DAPI (blue). Scale bar = 10 μm.

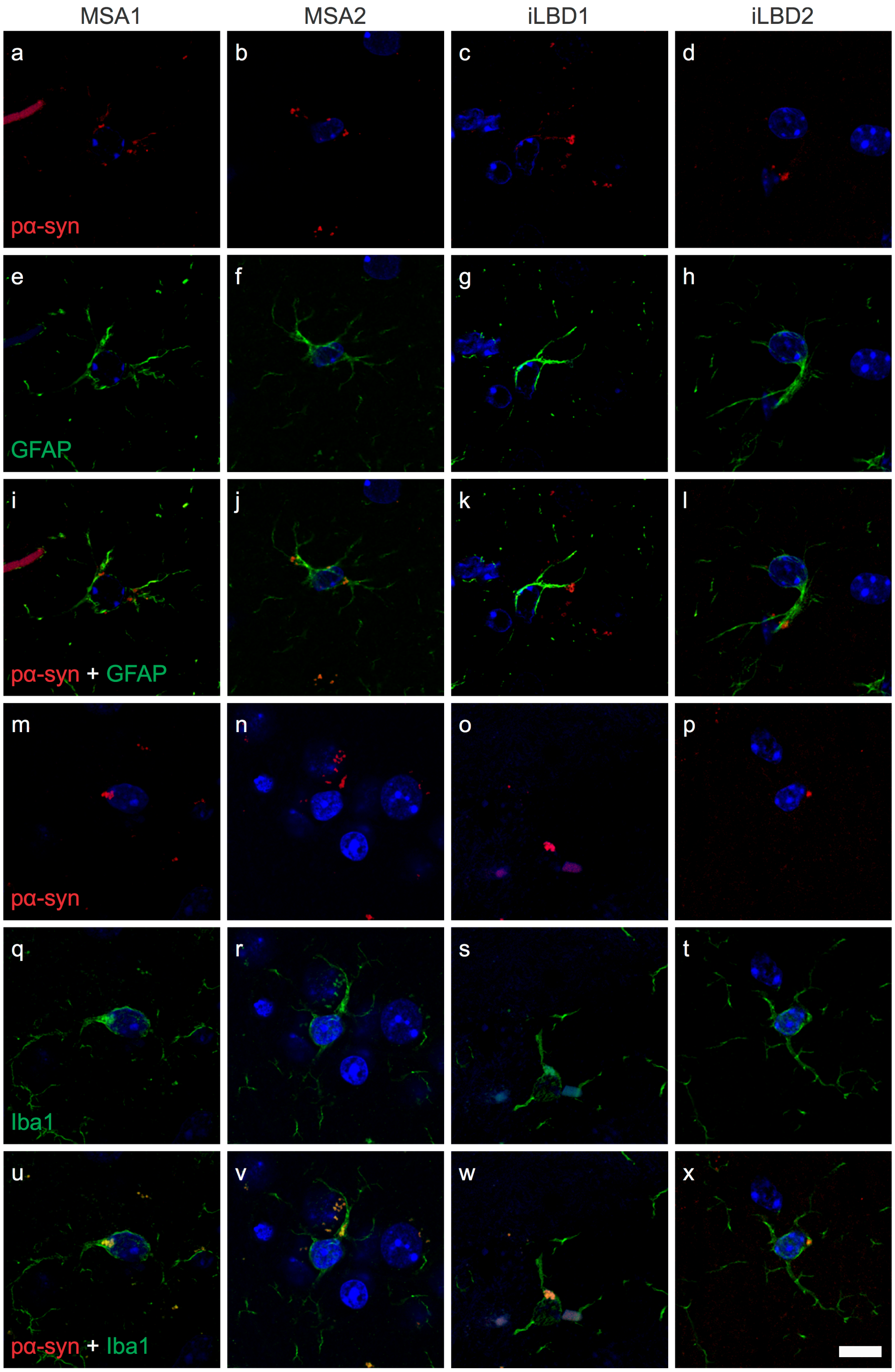

MSA1
MSA2
iLBD1
iLBD2
a
b
c
d
pα-syn
e
f
g
h
GFAP
i
j
k
l
pα-syn + GFAP
m
n
o
p
pα-syn
q
r
s
t
Iba1
u
v
w
x
pα-syn + Iba1
