# Supplementary material for: Prion-like propagation of human brain-derived alpha-synuclein in transgenic mice expressing human wild-type alpha-synuclein
Source: Acta Neuropathol Commun. 2015 Nov 26;3:75. doi: 10.1186/s40478-015-0254-7 (PMC4660655; doi:10.1186/s40478-015-0254-7)
Supplement: Additional file 5: — Phosphorylated alpha-synuclein in inclusion bodies colocalizes with sequestosome-1/p62. (PDF 5612 kb) [file 40478_2015_254_MOESM5_ESM.pdf]

Additional file 5 Phosphorylated alpha-synuclein in inclusion bodies colocalizes with sequestosome-1/p62

Confocal imaging of brain sections of Tg(SNCA)1Nbm/J mice injected with brain extracts from MSA or probable iLBD cases shows that at 9 months post injection staining with the pSyn#64 antibody for phosphorylated alpha-synuclein (**a**-**d**) and staining for sequestosome-1/p62 (**e**-**h**) colocalize when merged (**i**-**l**). Occasionally, a few smaller sized aggregates of sequestosome-1/p62 could be detected that did not appear to colocalize with phosphorylated alpha-synuclein. Nuclei were stained with DAPI (blue). Scale bar = 10 µm.

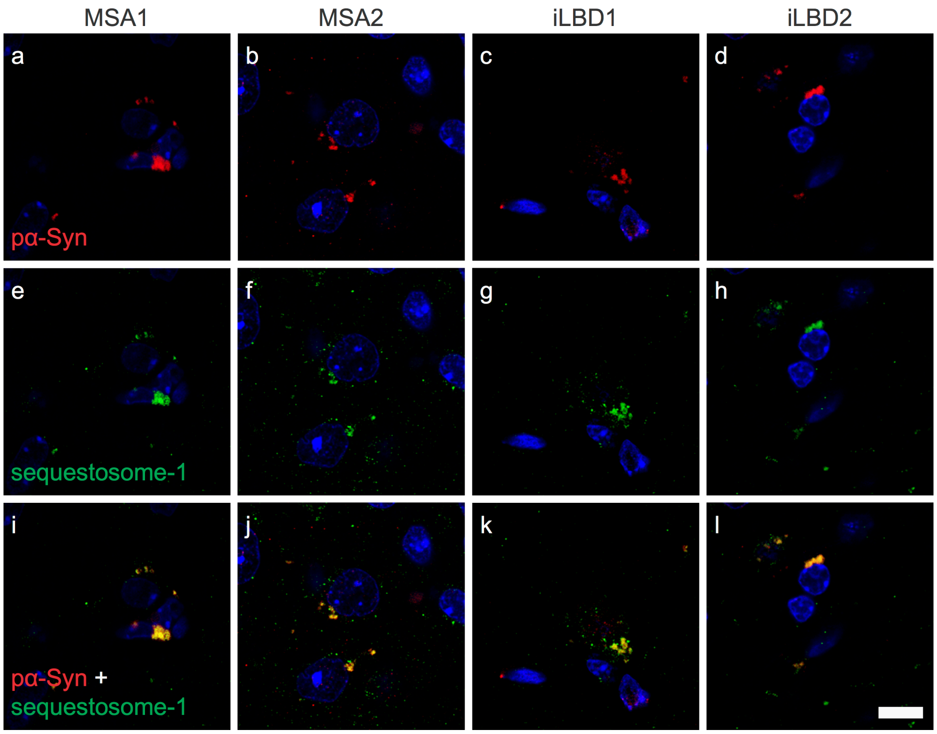
MSA1
MSA2
iLBD1
iLBD2
a
b
c
d
pα-Syn
e
f
g
h
sequestosome-1
i
j
k
l
pα-Syn + sequestosome-1
