# Supplementary material for: Prion-like propagation of human brain-derived alpha-synuclein in transgenic mice expressing human wild-type alpha-synuclein
Source: Acta Neuropathol Commun. 2015 Nov 26;3:75. doi: 10.1186/s40478-015-0254-7 (PMC4660655; doi:10.1186/s40478-015-0254-7)
Supplement: Additional file 6: — Reactive astrocytic gliosis in aged Tg(SNCA)1Nbm/J mice. (PDF 13304 kb) [file 40478_2015_254_MOESM6_ESM.pdf]

Additional file 6 Reactive astrocytic gliosis in aged Tg(SNCA)1Nbm/J mice

Immunohistochemical staining of mouse brain sections with antibodies against glial fibrillary acidic protein (GFAP) revealed mild reactive astrocytic gliosis within the hippocampus of all mice sacrificed at 9 months post injection, regardless whether brain extract from MSA or probable iLBD patients, or PBS was injected. Scale bar = 100 µm.

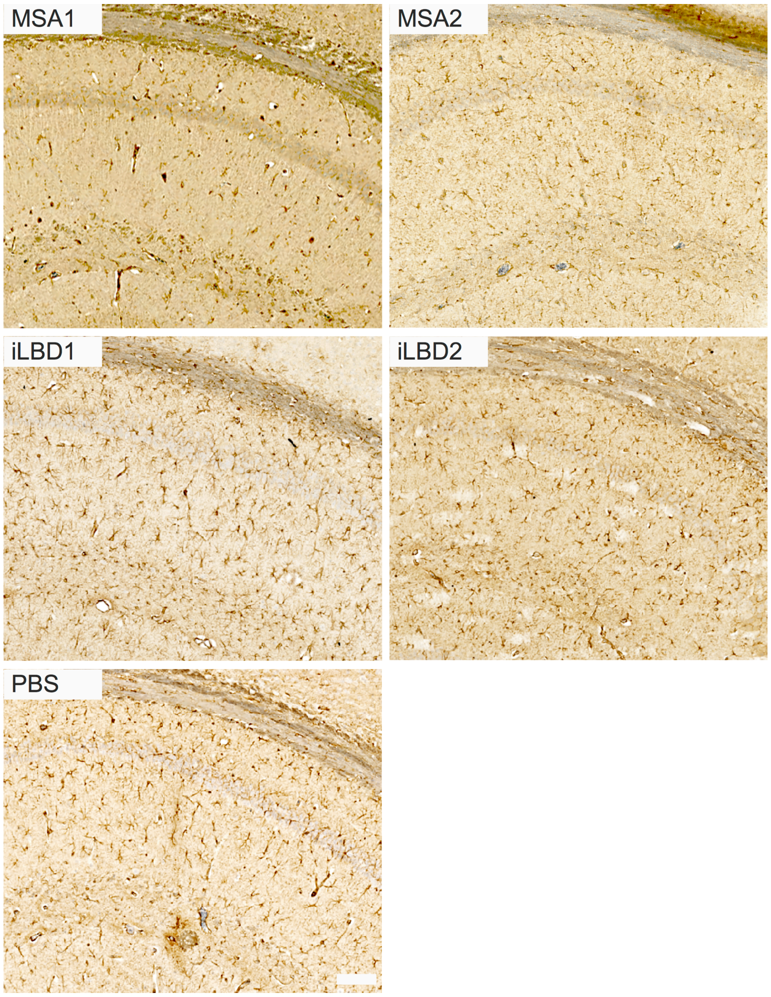

MSA1
MSA2
iLBD1
iLBD2
PBS
