# Supplementary material for: Prion-like propagation of human brain-derived alpha-synuclein in transgenic mice expressing human wild-type alpha-synuclein
Source: Acta Neuropathol Commun. 2015 Nov 26;3:75. doi: 10.1186/s40478-015-0254-7 (PMC4660655; doi:10.1186/s40478-015-0254-7)
Supplement: Additional file 7: — DAB-staining for Iba1 in aged Tg(SNCA)1Nbm/J mice. (PDF 14000 kb) [file 40478_2015_254_MOESM7_ESM.pdf]

Additional file 7 DAB-staining for Iba1 in aged Tg(SNCA)1Nbm/J mice

Brain sections of mice sacrificed at 9 months post injection were stained with antibodies against allograft inflammatory factor 1 (Iba1). Brains of mice injected with brain extracts from MSA or probable iLBD patients did not reveal increased microglial activation versus mouse brains injected with PBS. Unlike activated microglia with a more amoeboid morphology, the microglia observed here had the ramified morphology of resting microglia. Shown are cortical brain regions. Scale bar = 100 µm.

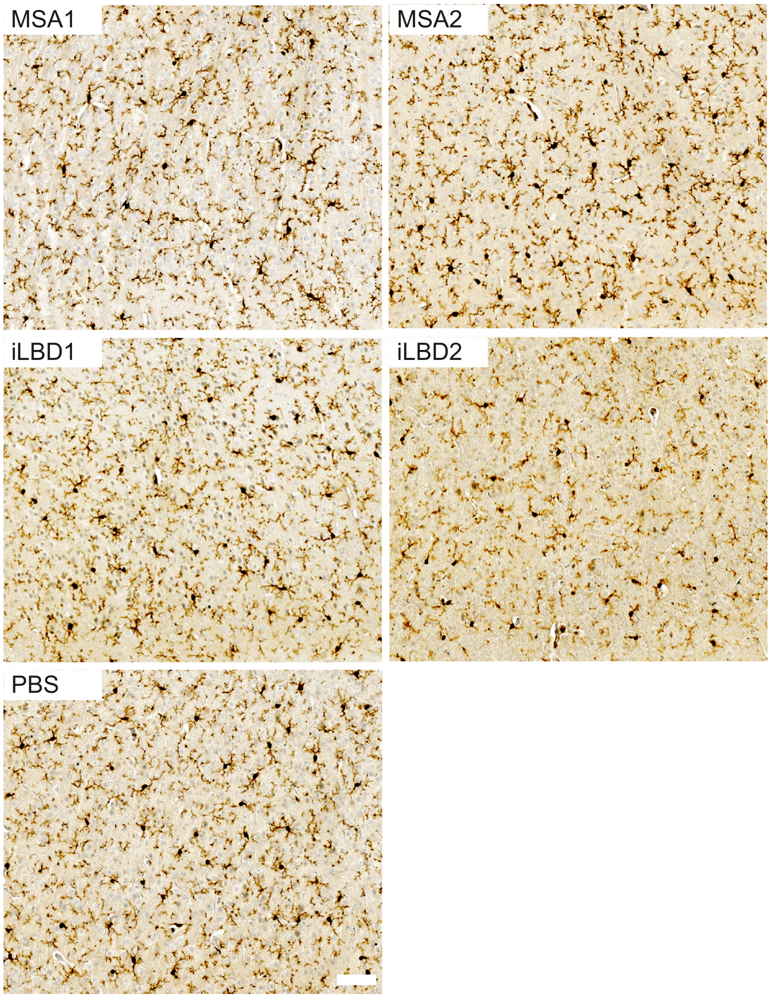
MSA1
MSA2
iLBD1
iLBD2
PBS
