# Supplementary material for: Prion-like propagation of human brain-derived alpha-synuclein in transgenic mice expressing human wild-type alpha-synuclein
Source: Acta Neuropathol Commun. 2015 Nov 26;3:75. doi: 10.1186/s40478-015-0254-7 (PMC4660655; doi:10.1186/s40478-015-0254-7)
Supplement: Additional file 8: — Brains of mice injected with brain extracts from MSA and probable iLBD cases did not accumulate phosphorylated alpha-synuclein that was sarkosyl insoluble. (PDF 398 kb) [file 40478_2015_254_MOESM8_ESM.pdf]

Additional file 8 Brains of mice injected with brain extracts from MSA and probable iLBD cases did not accumulate phosphorylated alpha-synuclein that was sarkosyl insoluble

Sarkosyl-insoluble fractions from brains of Tg(SNCA)1Nbm/J mice injected with brain extracts from MSA or probable iLBD cases or PBS were biochemically analyzed at 9 months post injection. Only a very faint band for monomeric phosphorylated alpha-synuclein became detectable with the 81A antibody after long exposure. Importantly, levels of phosphorylated alpha-synuclein were not elevated in animals injected with brain extracts from MSA or probable iLBD cases in comparison to animals injected only with PBS. Oligomeric species of phosphorylated alpha-synuclein were not detectable at all. Molecular sizes are shown in kilodalton.

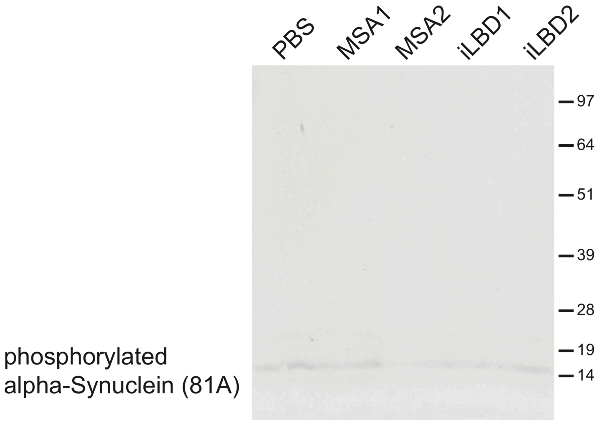
PBS
MSA1
MSA2
iLBD1
iLBD2
97
64
51
39
28
19
14
phosphorylated
alpha-Synuclein (81A)
